# Supplementary material for: Uptake of reproductive, maternal and child health services during the first year of the COVID-19 pandemic in Uganda: A mixed methods study
Source: PLOS Glob Public Health. 2023 Apr 20;3(4):e0001619. doi: 10.1371/journal.pgph.0001619 (PMC10118156; doi:10.1371/journal.pgph.0001619)
Supplement: Attachment — Submitted filename: Response to reviewers 27-01-2023.docx [file pgph.0001619.s001.docx]

27^th^ January 2023

Palash Chandra Banik, MPhil
Academic Editor, PLOS Global Public Health

**RESPONSE TO REVIEWERS**

Thank you for your positive review of our manuscript titled “Uptake of Reproductive, Maternal and Child Health Services during the First Year of the COVID-19 Pandemic in Uganda.” We have addressed both the journal and reviewers’ comments and are pleased to submit for continued review. Please see specific points below. Thank you for your consideration.

**Journal Requirements:**

1. Please amend your detailed Financial Disclosure statement. This is published with the article. It must therefore be completed in full sentences and contain the exact wording you wish to be published.

***Response:*** Revised.

a. State the initials, alongside each funding source, of each author to receive each grant.

***Response:*** Revised.

b. State what role the funders took in the study. If the funders had no role in your study, please state: “The funders had no role in study design, data collection and analysis, decision to publish, or preparation of the manuscript.”

***Response:*** This is now indicated under funding source.

c. If any authors received a salary from any of your funders, please state which authors and which funders.

2. Since your data is not available for proprietary reasons, please explain via email why the data is not available. Please also include the contact information for the third party organization that should be contacted should other researchers want to request access to this data and please include the full citation of where the data can be found. We also request that you verify with us via email that any researcher will be able to obtain the data set in the same manner that the you have obtained it. If you feel you are unwilling or unable to adhere to this policy, please explain your reasons by return email and your exemption request will be escalated to the editor for approval. Your exemption request will be handled independently and will not hold up the peer review process, but will need to be resolved should your manuscript be accepted for publication. One of the Editorial team will be in touch if they require more information.

***Response:*** We have added an updated statement on data access. It reads as follows. The dataset used for this paper is from the National Health Information System. This can be accessed on request to the permanent secretary, through the Director, Curative Services at the Ministry of Health, and through the Assistant Commissioners: Division of Health Information Management, email: [paul.mbaka@health.go.ug](mailto:paul.mbaka@health.go.ug), and the Division of Reproductive and Infant Health email: [richard.mugahi@health.go.ug](mailto:richard.mugahi@health.go.ug)

**Reviewer #1**:

1. I had a chance to review this study entitled, “Uptake of Reproductive, Maternal and Child Health Services during the First Year of the COVID-19 Pandemic in Uganda.” This study describes that use of reproductive health (RH), maternal, newborn and child health (MNCH) services in Uganda, like many resource-limited countries, is suboptimal. Reasons for this are complex; however, service-delivery factors such as availability, quality, staffing, and supplies, among others, contribute substantially to low uptake. The COVID-19 pandemic threatened to exacerbate many of the existing challenges to delivery and use of high-quality RH and MNCH services. We conducted a mixed methods study, combining secondary analysis of routine health management information system (HMIS) data with exploratory key informant interviews (KII) to examine changes in health service uptake over the course of the pandemic and to understand adaptations in service delivery that were implemented in response to the pandemic. We analyzed HMIS data for four services (family planning, facility-based deliveries, antenatal visits, and immunization for children by one year), and compared them across four time periods: pre-COVID-19, partial lockdown, total lockdown and post lockdown.
   I want to accept this study for publication. However, the authors need to revise it according to my suggestions. The title needs clarity with the design of the main study. I suggest authors revise their title with better and suitable words. See the below-recommended studies to improve your TTLE and Abstract quality.

***Response:*** Thank you so much for the review and the positive feedback about the quality of the manuscript. We have revised the title to include the design of the study. It now reads “Uptake of Reproductive, Maternal and Child Health Services during the First Year of the COVID-19 Pandemic in Uganda: A Mixed Methods Study.” See line 2.

2. Abstract: First, I have some suggestions for the authors to enhance the quality of this innovative study. Please write a high-quality abstract, as it is the main door of the study. I suggest authors add Graphical Abstract in a meaningful way to reflect the whole idea. The abstract should be in a structured format.

***Response:*** Thank you for the comment. We have separated the paragraph on methods to be distinct. It was mixed up in the introduction paragraph. The rest of the abstract is structured to cover introduction, methods, results and conclusions. We hope this is now clearer. We do not find an added advantage of a graphical abstract to this paper. We also have not seen any graphical abstract in papers in this journal.

3. Introduction section. This section needs improvement. Please read these studies, revise your abstract, and cite them in the introduction and literature part. Cite the suggested studies to improve the quality.
i. Su, Z., McDonnell, D., Wen, J., Kozak, M., Segalo, S., Li, X., Ahmad, J., Cheshmehzangi, A., Cai, Y., Yang, L., & Xiang, Y. T. (2021, Jan 5). Mental health consequences of COVID-19 media coverage: the need for effective crisis communication practices. Global Health, 17(1), 4. <https://doi.org/10.1186/s12992-020-00654-4>
ii. Azadi, N. A., Ziapour, A., Lebni, J. Y., Irandoost, S. F., & Chaboksavar, F. (2021). The effect of education based on health belief model on promoting preventive behaviors of hypertensive disease in staff of the Iran University of Medical Sciences. Archives of Public Health, 79(1), 69. doi:10.1186/s13690-021-00594-4
iii. Maqsood, A., Rehman, G., & Mubeen, R. (2021, 2021/11/01/). The paradigm shift for educational system continuance in the advent of COVID-19 pandemic: Mental health challenges and reflections. Current Research in Behavioral Sciences, 2, 100011. <https://doi.org/https://doi.org/10.1016/j.crbeha.2020.100011>
iv. Abbas, J. (2020). The Impact of Coronavirus (SARS-CoV2) Epidemic on Individuals Mental Health: The Protective Measures of Pakistan in Managing and Sustaining Transmissible Disease. Psychiatr Danub, 32(3-4), 472-477. https://doi.org/10.24869/psyd.2020.472

***Response:*** Thank you for the suggested articles. We politely note that we have reviewed the suggested articles, but did not find them related to this work directly to cite them in the introduction.

4. Literature. In the emergence of the COVID-19 pandemic, healthcare systems have faced a tremendous pressure worldwide. This pandemic has affected all lifestyles. The study investigated an interesting research topic. The study offers interesting information and provides useful insight. I suggest authors cite latest literature related to COVID-19 pandemic effects to support the study. The suggested studies are;
i. Zeidabadi, S., Abbas, J., Mangolian Shahrbabaki, P., & Dehghan, M. (2022). The Effect of Foot Reflexology on the Quality of Sexual Life in Hemodialysis Patients: A Randomized Controlled Clinical Trial. Sexuality and Disability, 41(1), 1-12. doi:10.1007/s11195-022-09747-x
ii. Rahmat, T. E., Raza, S., Zahid, H., Mohd Sobri, F., & Sidiki, S. (2022). Nexus between integrating technology readiness 2.0 index and students’ e-library services adoption amid the COVID-19 challenges: Implications based on the theory of planned behavior. J Educ Health Promot, 11(1), 50. doi:10.4103/jehp.jehp_508_21
iii. NeJhaddadgar, N., Ziapour, A., Zakkipour, G., Abolfathi, M., & Shabani, M. (2020, Nov 13). Effectiveness of telephone-based screening and triage during COVID-19 outbreak in the promoted primary healthcare system: a case study in Ardabil province, Iran. Z Gesundh Wiss, 1-6. <https://doi.org/10.1007/s10389-020-01407-8>
iv. Yoosefi Lebni, J., Moradi, F., Salahshoor, M. R., Chaboksavar, F., Irandoost, S. F., Nezhaddadgar, N., & Ziapour, A. (2020, Jul 2). How the COVID-19 pandemic effected economic, social, political, and cultural factors: A lesson from Iran. Int J Soc Psychiatry, 20764020939984. <https://doi.org/10.1177/0020764020939984>
v. Su, Z., McDonnell, D., Cheshmehzangi, A., Li, X., & Cai, Y. (2021). The promise and perils of Unit 731 data to advance COVID-19 research. BMJ Global Health, 6(4).

***Response:*** Thank you for the positive acknowledgement about the information that this study offers. Thank you also for the suggested articles. We have reviewed each article carefully, but could not find those relevant to be cited in this paper.

5. Methods and Results. Please read the suggested studies and improve your method design and result sections. Cite these studies in methods and results to support the literature
i. Li, Zhenhuan , Dake Wang, Kaifeng Duan, and R. Mubeen. 2021. "Social media efficacy in crisis management: Effectiveness of non-pharmaceutical interventions to manage the COVID-19 challenges." Front Psychiatry 12 (1099):626134. doi: 10.3389/fpsyt.2021.626134.
ii. Jiakui, C., Abbas, J., Najam, H., Liu, J., & Abbas, J. (2022). Green technological innovation, green finance, and financial development and their role in green total factor productivity: Empirical insights from China. Journal of Cleaner Production(381), 135131. doi:10.1016/j.jclepro.2022.135131
iii. Abbas, J., Aman, J., Nurunnabi, M., & Bano, S. (2019). The Impact of Social Media on Learning Behavior for Sustainable Education: Evidence of Students from Selected Universities in Pakistan. Sustainability, 11(6), 1683. <http://www.mdpi.com/2071-1050/11/6/1683>
iv. Aqeel, M., Rehna, T., Shuja, K. H. (2022). Comparison of Students' Mental Wellbeing, Anxiety, Depression, and Quality of Life During COVID-19's Full and Partial (Smart) Lockdowns: A Follow-Up Study at a 5-Month Interval. Front Psychiatry, 13, 835585. doi:10.3389/fpsyt.2022.835585
v. Farzadfar, F., Naghavi, M., Sepanlou, S. G., Saeedi Moghaddam, S., Dangel, W. J., Davis Weaver, N., . . . Larijani, B. (2022). Health system performance in Iran: a systematic analysis for the Global Burden of Disease Study 2019. The Lancet. doi:10.1016/s0140-6736(21)02751-3

***Response:*** Thank you for the suggested articles. However, we note that none of them is methodologically related to our study to be cited in the methods section. This is after a careful review of all the articles suggested.

6. Discussion is explained well. Check grammar errors in this section.
i. Aqeel, M., Raza, S., & Aman, J. (2021). Portraying the multifaceted interplay between sexual harassment, job stress, social support and employees turnover intension amid COVID-19: A Multilevel Moderating Model. Foundation University Journal of Business & Economics, 6(2), 1-17. doi:https://fui.edu.pk/fjs/index.php/fujbe/article/view/551
ii. Zhuang, D., Abbas, J., Al-Sulaiti, K., Fahlevi, M., Aljuaid, M., & Saniuk, S. (2022). Land-use and food security in energy transition: Role of food supply. Frontiers in Sustainable Food Systems, 6. doi:10.3389/fsufs.2022.1053031
iii. Schmidt, C. A., Cromwell, E. A., Hill, E., Donkers, K. M., Schipp, M. F., Johnson, K. B., . . . Hay, S. I. (2022). The prevalence of onchocerciasis in Africa and Yemen, 2000-2018: a geospatial analysis. BMC Med, 20(1), 293. doi:10.1186/s12916-022-02486-y
iv. Geng, J., Ul Haq, S., Ye, H., Shahbaz, P., Abbas, A., & Cai, Y. (2022). Survival in Pandemic Times: Managing Energy Efficiency, Food Diversity, and Sustainable Practices of Nutrient Intake amid COVID-19 Crisis. Frontiers in Environmental Science, 13, 945774. doi:10.3389/fenvs.2022.945774
v. Yu, S., Draghici, A., Negulescu, O. H., & Ain, N. U. (2022). Social Media Application as a New Paradigm for Business Communication: The Role of COVID-19 Knowledge, Social Distancing, and Preventive Attitudes. Frontiers in Psychology, 13. doi:10.3389/fpsyg.2022.903082

***Response:*** We have reviewed the discussion for grammatical errors, and didn’t find any, except a punctuation error. We stand to be corrected if there are that were observed by the reviewer. Regarding the articles, we did not use any of these articles, because none of them seems relevant to explain the results we found in this study.

7. Implications. Explain this section effectively. It needs a better presentation related to the study topic.

***Response:*** Thank you for this. We have the implications included in the discussion and conclusion sections. The author guidelines did not require a specific section on study implications.

8. Limitations. Discuss study’s limitations with a separate heading and discuss it briefly.

***Response:*** A limitation section is included according to journal guidelines (see the last paragraph of the discussion, starting at line 557.

9. Policy recommendations are not sufficient at this stage of the manuscript. The authors must add a separate section for policy recommendations in the conclusion section. Also, add some exciting limitations regarding political factors for future studies.

***Response:*** We discuss the importance of governmental guidelines on continued service provision, the potential impact of travel restrictions during COVID-19, and the importance of supporting community-based services and flexibility both within facilities and in health districts. We did not understand what the comment about “exciting limitations regarding political factors” meant in the context of this article. So, we did not make any “exciting limitations”, except for the study limitations as reported in the last paragraph of the discussion section. However, we do provide study limitations starting with line 557.

10. The conclusion section needs improvement and authors need to expand it as it will improve the quality of this study. The English level needs some improvement to reach a satisfactory level, specifically the grammar. It should sufficiently meet quality to reach scientific merit for publication. I recommend that the authors describe the study's scientific contribution to the existing body of knowledge in the discussion section. How does this study’s implications provide useful information for the scientific readership? I endorse this manuscript for publication after minor corrections, as suggested.

***Response:*** We have reviewed the entire manuscript and adjusted as recommended. We humbly request that if there are specific sections that need to be revised for grammar, it would be appreciated. We are unclear on which sections need to be improved in order for the manuscript to be of sufficient quality for publication. The discussion section details the contribution of the manuscript to the scientific literature.

**Reviewer #2:**

1. The manuscript was done in excellent way. It addressed the issue under study very well. The comparison made during periods of pre-covid, partial lack down, total lock down and post lock down was clearly shown and stated. It was compared, analyzed and discussed satisfactorily. The figures and tables are self-explanatory and easy to grasp and understand. The statistical analysis done was great. The conclusion derived matched the exact finding of the result. The recommendations forwarded agreed with the result and they will have great contribution. The study was ethically sound. Utilizing mixed methods of the quantitative and qualitative gives the paper better credit. I am really excited to see such high quality, well-prepared and well-done large-scale study. There are only few minor points I need to forward.

***Response:*** Thank you for the kind words. It is encouraging to know that the study is a useful contribution when published. We are grateful for the comments that you suggested to improve and have addressed them as indicated below.

2. Objective or the aim of the study should clearly be stated. Can you describe concisely what the research tries to achieve?

***Response:*** We have made the edit in the introduction, and hope that this is clearer now. The goal of this study was to track the impact of COVID-19 on the utilization of four family planning and maternal and newborn health services: family planning service visits, women delivering at health facilities, attendance of at least four antenatal care visits, and full immunization by one year of age. Additionally, the study sought to document adaptations utilized by the Government of Uganda and its partners to ensure the continuity of these health services during COVID-19. This has been clarified in the introduction section, starting at line 94.

3. Would you mention the study period for the qualitative study (KII) that you did?

***Response:*** Thank you for noting this oversight. The qualitative interviews were conducted in December 2020. This has been added to the manuscript. See line 203

4. Try to write out all abbreviations in full as introduced in the text the first time, such as WHO (line 59 and 121), ICT (line 308) and IUD (line 339).

***Response:*** This is done. Thank you.

5. The methodology was discussed in all-inclusive way.

***Response:*** Thank you.

6. However, it is unclear about how many health facilities were included in this study. Better also to explain the types of health facilities in which the secondary data were obtained; from hospitals, MCHs, clinics or health centers?

***Response:*** Thank you for this comment. We have added a statement to the methods section for further clarity. “All public and private facilities in the country providing MNCH services, that report in the eHMIS were considered. These included clinics, health centers and hospitals. Monthly reports for this paper are from 7,780 facilities.” (Line 134 to 137).

7. Can you explain health facilities in Uganda providing those services (Reproductive, maternal and child health services) at national level? Or at least general information about how and where those services are provided, so that the readers can understand and have full image of the scope of the study.

***Response:*** We have added this to the paper. The public sector includes the village health team that function as level I. These report to the health centres, and they offer some minimal family planning services and referrals for mothers as well as supporting mobilizations for vaccinations. The static public facilities are health center II, III, IV and hospitals. The private sector includes clinics and hospitals. The private include clinics and hospitals. The health services reported in this paper are offered at all levels with different levels of complexity. All the health centers and hospitals offer ANC, delivery services, immunization and family planning services, but with varied levels of complexity. (from line 110).

8. Were all health facilities in Uganda included in the study or they were just sampled? Would you briefly mention this? Let you be clear, specific and comprehensive. Can you very briefly describe about the administrative districts or states in Uganda too?

***Response:*** Thank you for noting this oversight. We have added an explanation from line 134 to clarify that “All public and private facilities in the country providing MNCH services, that report in the eHMIS were considered. These included clinics, health centers and hospitals. For the administrative districts, we have added a statement starting at line 110, that Uganda operates local governments that are called districts. These are subdivided into sub counties, parishes and villages. Within the districts, the public health facilities serve a health sub district which can include more than one sub county (Health center IV), sub county (health center III), parish (health center II and village (Village health teams). The district health officer is one of the technical heads at the district, overseeing the health services.

9. You mentioned that you did pretest. Can you explain where the pretest was done and if any modifications made? (Line 178)

***Response:*** Thank you for noting this important omission. We have added the pretest explanation to the manuscript, starting from line 205. ‘A pretest of the interview guides was conducted by the research team in three non-study districts; Kampala city, Kamuli and Jinja. All pretest interviews were conducted via phone, and audio recorded with consent, to assess feasibility of audio recording using the phone. After each interview, a template was completed to inform group learning around the scheduling of the interviews, the consenting process, duration, clarity of questions and any explicit respondent feedback on the interview experience. There were no major revisions to the content after the pretest, apart from reordering questions to improve the flow, defining the terms “pre-COVID, COVID and post- COVID” periods, and operationalizing the word “adaptations” especially for the VHTs.’  See starting at line

**Reviewer #3:**

1. This mixed method research has provided a good sense about the situation of essential services affected by Covid 19. In the method section, qualitative data analysis should be well explained.

***Response:*** Additional description of the qualitative analysis has been added. A thematic analysis was conducted using the transcripts of interviews. Data was coded using a excel-based matrix analysis structured around the deductive themes of inquiry in the interview guides and emerging themes from the interview data. Following coding and inner coder reliability checks, the data was summarized by theme across participant categories and illustrative quotes were selected to support the thematic results.

2. Please illustrate how privacy during interview and anonymity of the research participants were maintained.

***Response:*** We have added a statement to indicate what we did during data collection. “For the privacy of key informants, interviewers used ear buds and sat in a private place during the process to ensure no other person listened in. The key informants confirmed before the interview that they were in a private place and comfortable to talk.” (Line 218 to 221)

3. Data management also needs to be clear. The quantitative analysis of data is well narrated. But, qualitative data analysis requires further elaborations especially difference between high performing and low performing districts and the challenges.

***Response:*** The districts selected for qualitative interviews were selected based on the Ministry of Health’s ranking table which assigns a score for each district based on the pre-COVID-19 utilization of health services and health system resources available within each district. We have added a clarification on this score in the paragraph starting at lines 181 to 187 to clarify.

4. The discussion section well captured challenges that should be described well in results section.

***Response:*** Thank you.

5. Conclusion looks good.

***Response:*** Thank you.
